# High Performance Liquid Chromatography Separation of Epigenetic Cytosine Variants

**DOI:** 10.3390/mps1020010

**Published:** 2018-03-21

**Authors:** Caroline E. Roberts, Gregory M. Raner, Gary D. Isaacs

**Affiliations:** Biology and Chemistry Department, Liberty University, Lynchburg, VA 24515, USA; croberts85@liberty.edu (C.E.R.); graner@liberty.edu (G.M.R.)

**Keywords:** HPLC, methyl-cytosine, hydroxymethyl-cytosine, epigenetics

## Abstract

Epigenetic modifications enable cells to genetically respond to chemical inputs from environmental sources. These marks play a pivotal role in normal biological processes (e.g., differentiation, host defense and metabolic programs) but also contribute to the development of a wide variety of pathological conditions (e.g., cancer and Alzheimer’s disease). In particular, DNA methylation represents very stable epigenetic modification of cytosine bases that is strongly associated with a reduction in gene activity. Although High Performance Liquid Chromatography (HPLC) methodologies have been used to resolve methylated cytosine from unmodified cytosine bases, these represent only two of the five major cytosine analogs in the cell. Moreover, failure to resolve these other cytosine analogs might affect an accurate description of the cytosine methylation status in cells. In this present study, we determined the HPLC conditions required to separate the five cytosine analogs of the methylation/demethylation pathway. This methodology not only provides a means to analyze cytosine methylation as a whole, but it could also be used to more accurately calculate the methylation ratio from biological samples.

## 1. Introduction

Since the discovery of cytosine demethylation, hydroxymethylcytosine (hmC) has been identified as a possible “sixth base” due to its apparent involvement in gene expression [1], especially in neuronal tissue where hmC levels appear to be the highest [2]. These findings have prompted the need for methodologies that are able to distinguish between highly similar cytosine analogs. In the past, scientists have used high performance liquid chromatography (HPLC) to analyze global cytosine methylation, which overlooked three of the five cytosine variants involved in the demethylation cycle. As the outlook on epigenetics shifts in order to accept a wider array of potential modifiers (i.e., hmC), HPLC methodologies should do likewise if possible.

**Figure 1:**
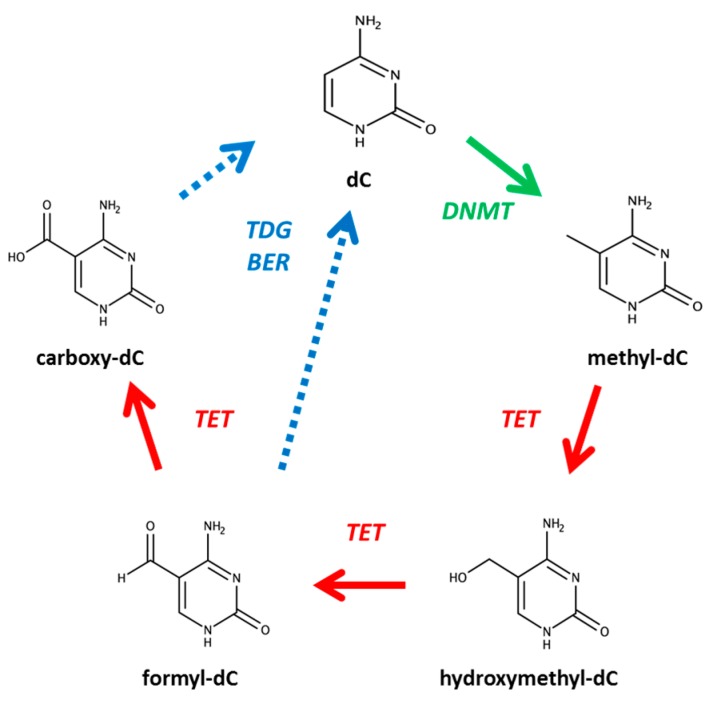
Cytosine modifications. Deoxycytidine (dC) is modified by DNA methyltransferases (DNMT) and ten-eleven translocation (TET) proteins. Recycling reactions by thymine DNA glycosylase (TDG) or base excision repair (BER) enzymes returns modified cytosine to its unmodified state.

High performance liquid chromatography is a frequently used method for the quantification of DNA bases in organismal samples [3]. For example, several recent studies have demonstrated the effectiveness of this method in the identification and quantitative analysis of cytosine and methylcytosine (mC) in several different biological samples [4,5,6,7,8]. Recently, it has been determined that cytosine, following methylation, can undergo demethylation through the action of the ten-eleven translocation (TET) family of enzymes [9,10,11]. In this process, mC is sequentially oxidized to hmC, formylcytosine (fC) and carboxycytosine (caC) (Figure 1). This cycle ultimately leads to the regeneration of unmodified cytosine. Discovery of this demethylation pathway has led to the identification of 5hmC as a possible epigenetic modifier [2,12,13,14,15,16,17] and 5fC and 5caC as potential regulators in the regeneration of cytosine. Due to this, one must differentiate between the five variants of cytosine when determining methylation status. 

Many studies using HPLC alone for the examination of global cytosine methylation have failed to address the possible presence of all analogs of cytosine [7]. Such studies should be viewed with caution as they could present misleading data concerning the methylation (or modification) ratio of cytosine since they do not focus on all modified variants compared to unmodified cytosine. Some have used HPLC coupled with mass spectroscopy (MS) [18,19] to successfully separate and quantify all cytosine analogs involved in the demethylation pathway, but this process requires more specialized instrumentation than a simple HPLC with ultraviolet absorbance (UV) detection. One study has been successful using HPLC alone to resolve cytosine variants relative to a uracil standard [20], although the run time for this method was 25 min and the analysis did not compare the cytosine variants to unmodified cytosine. Therefore, a more efficient HPLC method that does not require MS is needed to analyze cytosine variants relative to unmodified cytosine. This would greatly benefit those seeking to determine genomic methylation status of organismal DNA samples. 

## 2. Materials and Methods

All chemicals were of analytical reagent grade. Deionized water (18.2 MΩ·cm) obtained from a Millipore (Billerica, MA, USA) Milli-Q system was used throughout the experiment. The deoxynucleoside standards (2′-deoxycytidine, 5-methyl-2′-deoxycytidine, 5-hydroxymethyl-2′-deoxycytidine, 5-formyl-2′-deoxycytidine, 5-carboxy-2′-deoxycytidine) were obtained from Jena Bioscience (Jena, Germany). Standard stock solutions were prepared by dissolving the commercial nucleosides in deionized water at concentrations of 2–10 mg·Ml^−1^. Working standard solutions were prepared as needed by diluting stock solutions with deionized water. HPLC grade methanol from Fisher Scientific (Hampton, NH, USA) was used as mobile phase A. Ammonium phosphate monobasic (Fisher Scientific), phosphoric acid (Fisher Scientific) and sodium hydroxide (Sigma; St. Louis, MO, USA) were used in the preparation of mobile phase B (phosphoric acid and sodium hydroxide were used to adjust the phosphate buffer to the appropriate pH). Identification of each peak was performed by running individual standards or by running a mixture of these standards at different concentrations.

Chromatographic separations were performed using an Agilent 1260 Infinity II series system (Agilent Technologies; Santa Clara, CA, USA), which consisted of an in-line degasser, 100-well autosampling and diode array detection that operated with a standard binary pump. Acquisition of data and subsequent calculations were performed using ChemStation software provided by Agilent. Two reversed phase HPLC columns were tested in the optimization of a technique to separate the deoxynucleoside standards; an Agilent C18 (50 × 3 mm, 1.8 μm particle size) and a Phenomenex (Torrance, CA, USA) Luna Phenyl Hexyl (150 × 4.6 mm, 5 μm particle size). Experimental operating conditions used throughout protocol optimization are summarized in Table 1. The final operating conditions used to resolve all of the deoxynucleoside standards are summarized in Table 2.

## 3. Results and Discussion

### 3.1. Resolution of Cytosine Analogs Using C18 Column

**Figure 2:**
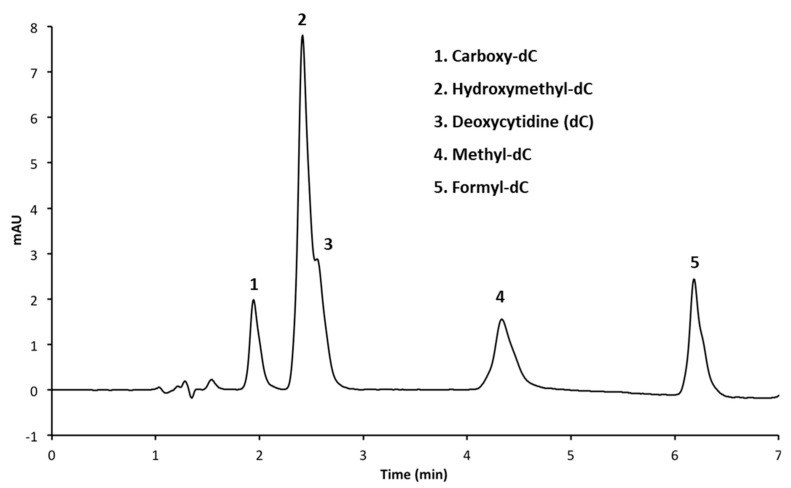
HPLC chromatogram of five deoxycytidine analogs on a C18 column with phosphate buffer (pH = 4).

Initially, we attempted to resolve the five cytosine standards using an HPLC protocol similar to one previously developed for the separation of cytosine from mC [7]. While this provided adequate separation of caC, mC and fC, it did not significantly resolve the hmC peak from the unmodified cytosine standard (Figure 2). It is interesting to note that hmC demonstrated significantly more absorption at the detection wavelength than unmodified cytosine when the same concentrations were loaded (Figure 2, peaks 2 and 3).

### 3.2. Resolution of Cytosine Analogs Using Phenyl Hexyl Column

#### 3.2.1. Performing HPLC Runs at a pH of 4.0

**Figure 3:**
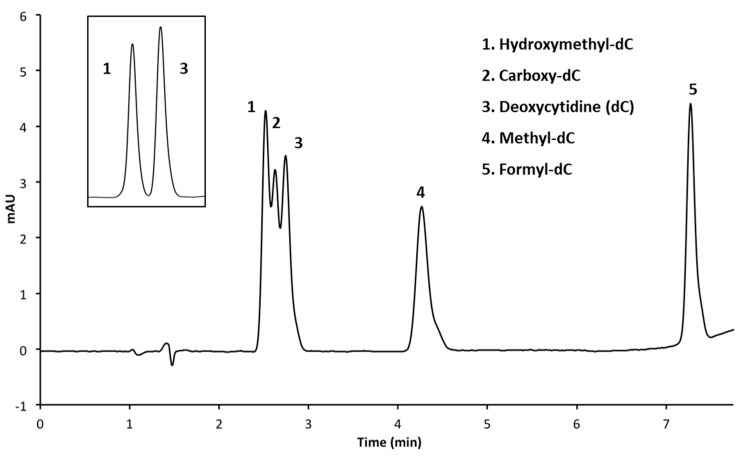
HPLC chromatogram of five deoxycytidine analogs on a phenyl hexyl column with phosphate buffer (pH = 4). Insert of only hmC and cytosine under the same operating conditions demonstrates adequate separation of these molecules.

In order to provide an alternative for C18 selectivity, we ran the cytosine standard mixture over a phenyl hexyl HPLC column, aiming to resolve the polar hmC from the slightly less polar cytosine. The phenyl hexyl column separated the mC and fC similar to the C18 run but produced a triple peak with mild resolution composed of the hmC, caC and cytosine standards (Figure 3). The purpose for using the phenyl hexyl column was to separate the hmC from the unmodified cytosine, which was demonstrated when only these two standards were used (Figure 3 inset graph). It is important to note that the amount of hmC used in these runs was approximately half the concentration that was used in Figure 2 since we previously determined the sensitivity of detection for hmC. Although these HPLC conditions could resolve the hmC and cytosine standards, the addition of caC produced an overlapping elution that prevented adequate resolution of those peaks.

#### 3.2.2. Performing HPLC Analysis at a pH of 7.0

**Figure 4:**
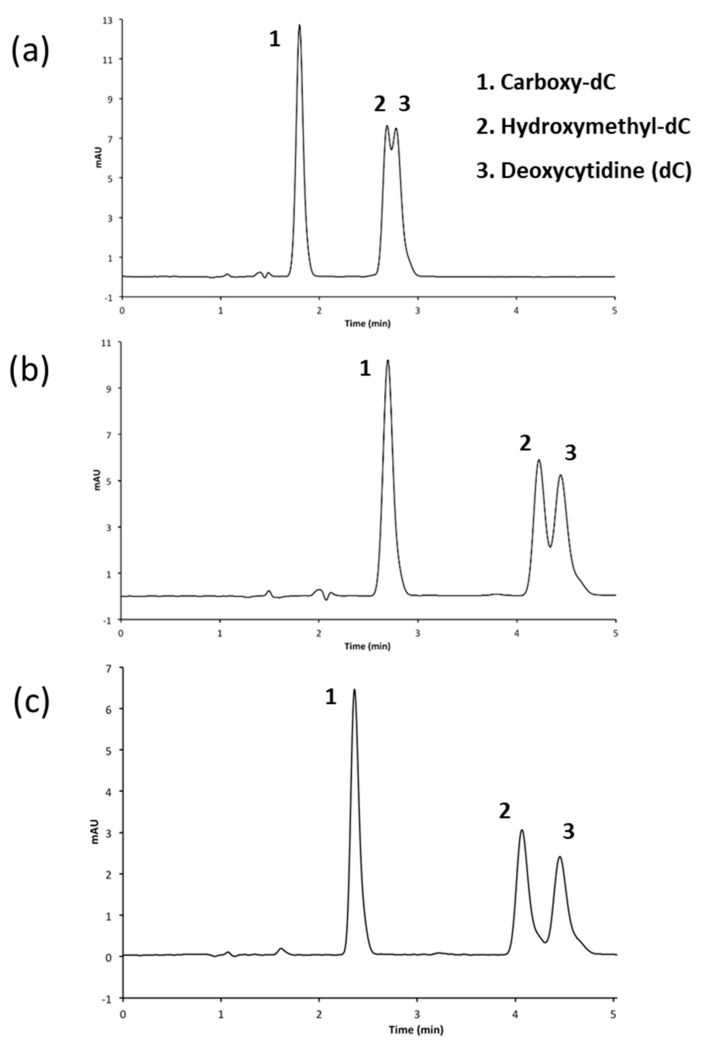
HPLC chromatogram of three deoxycytidine analogs on a phenyl hexyl column with phosphate buffer (pH = 7). Subsequent runs have decreasing methanol gradients for the first eight minutes of running time: (**a**) 5%; (**b**) 3%; and (**c**) 1%.

The phenyl hexyl column caused a shift in the caC elution time, causing it to overlap with the resolved hmC and cytosine peaks. After this, the standard mix was analyzed on the phenyl hexyl column at a neutral pH in an attempt to ionize the caC and shift its elution to much earlier in the run. The standard cytosine mixture ran in a similar fashion to the C18 run at a pH of 4.0 with only slightly better resolution between the hmC and cytosine peaks (Figure 4a).

#### 3.2.3. Adjusting Methanol Concentration to Improve Resolution

**Figure 5:**
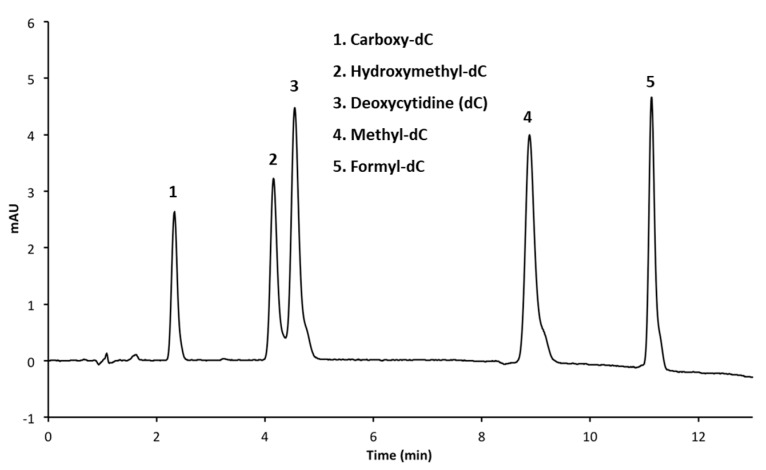
HPLC chromatogram of all five deoxycytidine analogs on a phenyl hexyl column with phosphate buffer (pH = 7).

The methanol concentration of the mobile phase was reduced from 5% in order to increase the retention time of the analytes on the phenyl hexyl column and improve the separation between the hmC and cytosine. Decreasing the methanol concentration to 3% increased the retention time by approximately 1 min, which allowed for increased resolution between the hmC and cytosine peaks. Decreasing the methanol to 1% improved the resolution even further (Figure 4a–c). This longer retention time still permitted the resolution of all five cytosine analogs in less than 12 min, which is approximately twice as fast as the previously published protocol for the separation of cytosine variants from uracil [20] (Figure 5).

## 4. Conclusions

Based on the results of this study, HPLC can be used to separate and quantify all five of the cytosine variants simultaneously if they are present in their single nucleotide, dephosphorylated forms. In addition, the findings suggest that the previous studies using HPLC to quantify cytosine and mC might need to be re-examined due to the high likelihood that cytosine peaks also contain hmC. This is a critical finding that should also be applied to future analyses, especially neuronal studies, in order to avoid masking the presence of hmC in organismal DNA samples. To that end, the HPLC method described in this technical note can be used as an effective first step in the development of an epigenetic approach to enhance our understanding of cytosine modifications in an economical and efficient manner.

## Figures and Tables

**Table 1:** Methods used in the optimization of an HPLC technique for the separation of deoxycytidine analogs.

Figure	Column	Mobile Phases	Elution (%, *v*/*v*)	Flow Rate mL/min
2	Agilent C18 (50 × 3 mm, 1.8 μm)	A: CH_3_OH B: 50 mM NH_4_H_2_PO_4_ (pH 4.0) C: H_2_O	3 min 5% A, 15% B 6 min 20% A, 15% B 6.05 min 30% A, 15% B 9 min 30% A, 15% B 9.05 min 5% A, 15% B 13 min 5% A, 15% B	1.4
3	Luna Phenyl Hexyl (150 × 4.6 mm, 5 μm)	A: CH_3_OH B: 50 mM NH_4_H_2_PO_4_ (pH 4.0) C: H_2_O	4 min 5% A, 15% B 6 min 20% A, 15% B 6.05 min 30% A, 15% B 9 min 30% A, 15% B 9.05 min 5% A, 15% B	1.4
3 (insert)	Luna Phenyl Hexyl (150 × 4.6 mm, 5 μm)	A: CH_3_OH B: 50 mM NH_4_H_2_PO_4_ (pH 4.0) C: H_2_O	8 min 5% A, 15% B 11 min 20% A, 15% B 11.05 min 30% A, 15% B 16 min 30% A, 15% B 16.05 min 5% A, 15% B	1.4
4a	Luna Phenyl Hexyl (150 × 4.6 mm, 5 μm)	A: CH_3_OH B: 50 mM NH_4_H_2_PO_4_ (pH 7.0) C: H_2_O	8 min 5% A, 15% B 11 min 20% A, 15% B 11.05 min 30% A, 15% B 16 min 30% A, 15% B 16.05 min 5% A, 15% B	1.4
4b	Luna Phenyl Hexyl (150 × 4.6 mm, 5 μm)	A: CH_3_OH B: 50 mM NH_4_H_2_PO_4_ (pH 7.0) C: H_2_O	8 min 3 % A, 15% B 11 min 20% A, 15% B 11.05 min 30% A, 15% B 16 min 30% A, 15% B 16.05 min 3% A, 15% B	1.0
4c	Luna Phenyl Hexyl (150 × 4.6 mm, 5 μm)	A: CH_3_OH B: 50 mM NH_4_H_2_PO_4_ (pH 7.0) C: H_2_O	8 min 1% A, 15% B 11 min 20% A, 15% B 11.05 min 30% A, 15% B 16 min 30% A, 15% B 16.05 min 1% A, 15% B	1.4

**Table 2:** Operating conditions of optimized protocol (corresponding Figure: 5).

Column	Luna Phenyl Hexyl (150 × 4.6 mm, 5 μm)		
Mobile phases	A: Methanol		
	B: 50 mM ammonium phosphate (pH of 7.0)		
	C: Deionized water		
Gradient elution	Time (min)	%A	%B
	0	1	15
	6	1	15
	14	30	15
	16	30	15
	16.05	100	0
	18	100	0
	18.05	1	15
	21	1	15
Injection volume	50 μL		
Actual injection	40 μL		
Flow rate	1.4 mL·min^−1^		
Detection	Diode-Array Dectector 1 A, Signal = 280 nm Reference = 360 nm		
